# Dr. M. S. Dean

**Published:** 1882-03

**Authors:** 


### OBITUARY.

*Dr. M. S. Dean*, a brief notice of whose death, on the 28th of January, 1882, appeared in the February number of this JOURNAL, was born in Pittsfield, Vermont, in 1825. Dr. Deans' first occupation was teaching, which he continued until 1843 when he studied medicine in Ogdensburg, New York, and afterwards dentistry with Dr. D. C. Ambler. His first location as a dental practitioner was Dundas, Canada, then Milan, Ohio, where he remained until 1852, at which time he removed to Marshall, Michigan. In 1864 he removed to Chicago where he continued in active practice until his death. Dr. Dean was one of the most popular and eminent practitioners of dentistry in this country, and was honored with prominent positions in his State and the National Dental Associations. He was elected President of the Illinois State Dental Society, and twice elected to the same office in the Chicago Dental Society. For five years he was the Secretary and Chairman of the Publication Committee of the American Dental Association, and in 1875 was elected President of that body. Dr. Dean contribnted many papers of interest and value to the different societies and to the dental journals, and within a few years past translated Legro's and Magitot's treatise on "The Dental Follicle," with the results of his own researches, which made a valuable addition to the literature of Dental Science. As a citizen he was universally respected; and among his colleagues was regarded as a man of unusually clear perceptions and sound judgement. His intercourse with fellow practitioners was noted for kindness and consideration, and in his death the dental profession has lost one of its best and most accomplished members.

---

